# A Rare Case of Hernia Incarceration Under the Closed Port-Site Fascia After Robot-Assisted Laparoscopic Radical Cystectomy: Insights and Management Strategies

**DOI:** 10.7759/cureus.34915

**Published:** 2023-02-13

**Authors:** Shinro Hata, Shunsuke Nakashima, Mayuka Shinohara, Toshitaka Shin, Hiromitsu Mimata

**Affiliations:** 1 Department of Urology, Faculty of Medicine, Oita University, Yufu, JPN

**Keywords:** port-site hernia, urinary bladder ca, robot-assisted surgery, cystectomy, hernia

## Abstract

Most cases of port-site hernia were due to inadequate fascial closure of the port site. We experienced a rare case of hernia incarceration under the closed port-site fascia despite adequate closure of the fascia after robot-assisted laparoscopic radical cystectomy. In this case, the small intestine was incarcerated between the transversus abdominis and oblique abdominal muscles from the 12-mm trocar site for the assistant. We inserted forceps to release the incarceration, and the fascia and peritoneum of the port site were closed using a trocar site closure device under laparoscopy. We considered that all-layer suturing, including peritoneum and inner and outer oblique fascia suturing, was necessary for port-site closure, especially in patients with obesity, because hernias can occur with fascial closure alone.

## Introduction

In recent years, robot-assisted laparoscopic surgery has become widely used as a standard procedure in the field of urology, enabling less invasive treatment [1,2]. Port-site hernia is a well-known but rare complication of laparoscopic procedures [3-5]. Recent studies on the occurrence of port-site hernia in robot-assisted urological surgery revealed an incidence of 0.66% [6]. On the other hand, the incidence of incisional hernia in robot-assisted laparoscopic surgery is reported to be higher than that in open surgery [7,8]. However, the true incidence is suspected to be greater than reported because of asymptomatic patients not seeking medical attention, diagnostic failures, or delays. Port-site hernia in robot-assisted laparoscopic surgery is mainly caused by inadequate fascial closure, and other causes are rare. Here, we report a case of subfascial hernia incarceration despite adequate fascial closure after robot-assisted laparoscopic radical cystectomy (RARC). 

## Case presentation

**Figure 1:**
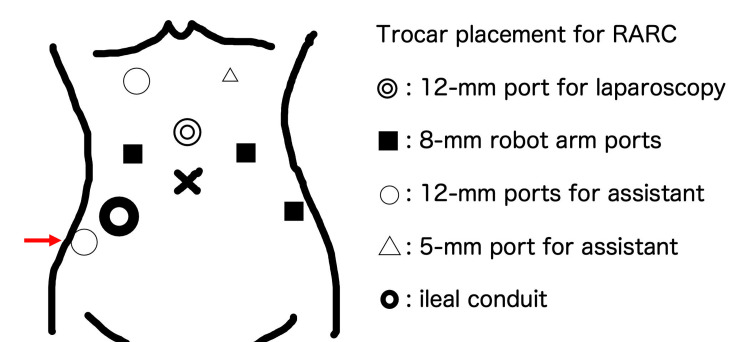
Trocar placement for RARC RARC: Robot-assisted laparoscopic radical cystectomy, Image Credits: Shinro Hata

A 70-year-old Japanese man (height, 164.5 cm; weight, 65.7 kg; body mass index, 24.3 kg/m2) was diagnosed with Bacillus Calmette-Guérin refractory bladder cancer (cTis N0 M0). He underwent RARC with an intracorporeal ileal conduit-urinary diversion and extended pelvic lymphadenectomy. The trocar placement is shown in Figure 1.

**Figure 2:**
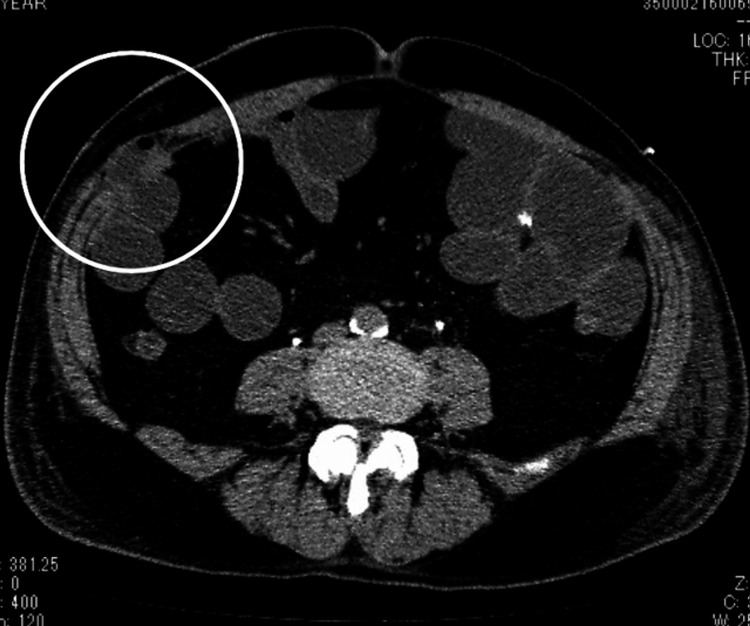
Abdominal computed tomography Computed tomography showed the small intestinal incarceration in the right abdomen.

He had no significant past surgical history. The total operative and console times were 722 and 593 min, respectively. The estimated blood loss volume was 240 ml. We closed the fascia of all the ports by using an absorbable suture. The early postoperative period was uneventful. The patient resumed oral intake on the seventh day after the surgery. Eight days after the surgery, he complained of severe abdominal pain and nausea. Abdominal computed tomography revealed herniation of the small intestine from the 12-mm trocar site for the assistant (Figure 2).

**Figure 3:**
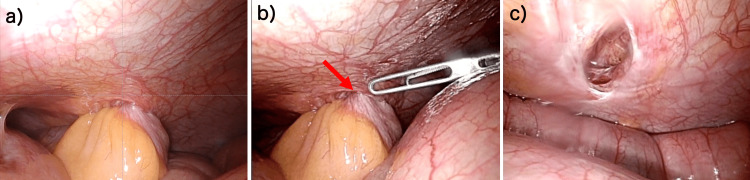
Laparoscopic view of incarcerated small intestine a) The small intestine had been incarcerated. b) The incarcerated small intestine was replaced with forceps. The arrow shows the incarcerated intestine. c) The peritoneum was opened, but the fascia was completely closed.

An emergency explorative laparotomy revealed that the small intestine was partially prolapsed from the 12-mm trocar site (Figure 3A).

We placed a 5-mm port at the first arm port for RARC and inserted forceps to release the incarceration (Figure 3B). We observed the wound in which the small intestine had been incarcerated and found that the peritoneum was opened, but the fascia was completely closed (Figure 3C). A laceration of the peritoneum and transversus abdominis fascia at the insertion site of the 12-mm port for the assistant was observed. This site became a hernia orifice, and the small intestine was incarcerated (Figure 3C). We diagnosed an early postoperative port-site hernia due to the formation of a space between the transversus abdominis and oblique abdominal muscles at the 12-mm port site for the assistant, into which the small intestine was entrapped. The peristalsis and coloration of the incarcerated small intestine were good. The fascia and peritoneum of the port site were closed using a trocar site closure device under laparoscopy. The patient was discharged 23 days after the RARC, and his clinical course was uneventful throughout the follow-up period. The final pathological diagnosis was pTis N0 M0.

## Discussion

We experienced a rare case of hernia incarceration under the closed port-site fascia despite adequate closure of the fascia. The occurrence of port-site hernias is related to procedural and patient factors. The former includes trocar type, diameter, insertion position, presence or absence of fascial closure, thread type, and insufflation pressure, while the latter includes age, body mass index, and presence of wound infection [9,10]. In the placement of an assistant port, it is common to use a blade-less trocar to separate the oblique abdominal muscles without cutting them, and theoretically, after port removal, the fascia will spontaneously heal, the wound will close, and herniation is unlikely to occur [4]. In this case, the small intestine was incarcerated between the transversus abdominis and oblique abdominal muscles. Such incarceration is due to the fragile condition of each layer and the bond between layers that make up the abdominal wall tissue. Conversely, solid tissues would provide no space for the intestine to be incarcerated in.

When re-operation is performed after the development of a port-site hernia, either an open or laparoscopic procedure is chosen [11]. In this case, the laparoscopic procedure was chosen, and the location and status of the hernia orifice were determined from within the abdominal cavity. Furthermore, observation of the coloration of the small intestine and secure suture closure of the peritoneum, muscles, and fascia were possible.

We considered that all-layer suturing, including peritoneum and inner and outer oblique fascia suturing, was necessary for port-site closure, especially in patients with obesity, because hernias can occur with fascial closure alone. A trocar site closure device under laparoscopy is also useful because it allows for the secure closure of the port site.

## Conclusions

We report a case of port-site hernia after RARC. This complication occurs at a certain rate even in the absence of applicable procedural and patient factors, and efforts should be made to employ techniques to reduce it, and to detect it early. The laparoscopic procedure is also useful for port-site hernia repair after robotic surgery and should be the first technique attempted.
